# Identifying Undiagnosed High-Risk Suicidality Cases by Matching Patients With a Similar Comorbidity Burden: Retrospective Observational Study

**DOI:** 10.2196/81499

**Published:** 2026-03-12

**Authors:** Louisa Bode, Rena Xu, Matthew Garber, Kenneth D Mandl, Andrew J Mcmurry

**Affiliations:** 1 Peter L. Reichertz Institute for Medical Informatics, TU Braunschweig and Hannover Medical School, Hannover, Germany; 2 Computational Health Informatics Program, Boston Children's Hospital, 300 Longwood Ave, Boston, MA, 02115, United States, 1 (617) 355-6000; 3 Department of Surgery, Harvard University, Boston, MA, United States; 4 Department of Pediatrics, Harvard Medical School, Boston, CA, United States

**Keywords:** pediatrics, mental disorders, suicidal ideation, self-injurious behavior, propensity score, medical informatics, comorbidity, electronic health records, Diagnostic and Statistical Manual of Mental Disorders, DSM

## Abstract

**Background:**

Suicide is the second leading cause of death for children and adolescents aged 6 to 18 years. Pediatric suicidality is underreported, which poses significant challenges for effective intervention and prevention strategies. Identifying populations at risk of suicidality can provide critical benefits in terms of study cohort selection, prevalence estimation, and clinical resource allocation.

**Objective:**

This study sought to (1) measure the prevalence of mental health comorbidities in pediatric suicidality and (2) identify undiagnosed high-risk suicidality cases by matching them with patients with a similar mental health comorbidity burden.

**Methods:**

Electronic health record data from a large academic pediatric hospital in Boston, Massachusetts, were analyzed for patients aged 6 to 18 years presenting to the emergency department between June 1, 2016, and June 1, 2022. Suicidality cases were defined using *International Classification of Diseases, 10th Revision* (*ICD-10*) codes for 3 suicidality subtypes: suicidal ideation, self-harm, and suicide attempt. Comorbidities of suicidality were calculated as the conditional probability of *ICD-10* code pairs. After multiple hypothesis corrections, statistically significant comorbidities and patient encounter demographics were input as covariates into a propensity score matching (PSM) model. The accuracy of the PSM model was validated against chart review by 2 independent subject matter experts.

**Results:**

In total, 2.9% (2638/90,980) of emergency department encounters met an *ICD-10*–based case definition of suicidality during the study period. The prevalence of suicidality by subtype was 2.5% (2275/90,980) for ideation, 1.1% (1030/90,980) for self-harm, and 0.2% (177/90,980) for suicide attempt. Suicidality prevalence was more common for the female sex (1825/43,929, 4.2%) than for the male sex (813/47,045, 1.7%). Comorbidities of suicidality were statistically significant for 55 frequently co-occurring *ICD-10* codes. Nearly half of these comorbidities (26/55, 47.3%) were not present in the *Diagnostic and Statistical Manual of Mental Disorders, Fifth Edition*, and nearly a quarter (12/55, 21.8%) consisted of *ICD-10* codes for accidental rather than intentional self-harm. Increased probability of suicidality was observed for patients with personality disorder (84/190, 44.2%), gender dysphoria (143/333, 42.9%), bipolar disorder (162/448, 36.2%), depression (1791/5426, 33%), and schizophrenia spectrum disorders (133/411, 32.4%). On the basis of gold-standard chart review, 53.4% of propensity-matched noncases were unrecognized cases of suicidality.

**Conclusions:**

PSM using comorbidity profiles is an effective approach for identifying suicidality cases that lack *ICD-10* codes for suicidality.

## Introduction

Suicide is the second leading cause of death among children and adolescents aged 6 to 18 years in the United States [1], underscoring a major public health issue. The prevalence of cases of pediatric suicidal ideation, self-harm, and attempts in emergency departments (EDs) has dramatically increased [2-4]. However, many at-risk children and adolescents may never receive a formal diagnosis of suicidality (ie, due to variability in coding practices or stigma-related nondisclosure). Using *International Classification of Diseases, 10th Revision* (*ICD-10*)–based case definitions for prevalence estimation or cohort selection may lead to misclassifications or incomplete capture of cases [5-8], ultimately underestimating the true prevalence of suicidal behavior among children and adolescents. Additionally, the standard *Diagnostic and Statistical Manual of Mental Disorders, Fifth Edition* (*DSM-5*) [9], which is used to guide hospital coding, may not comprehensively identify relevant psychiatric comorbidities.

Mental health comorbidity plays a crucial role in suicide risk [10-12]. Suicidality in youth arises from a complex interplay of emotional, cognitive, and environmental changes during development. Recent work shows that rapid neural and behavioral maturation during adolescence can increase vulnerability to suicidal thoughts and behaviors [13]. Psychopathological mechanisms such as impaired emotion regulation, heightened stress reactivity, and early exposure to adverse experiences further interact with these developmental processes to increase suicide risk [14]. Because of this critical interplay, understanding the relationship between certain comorbidity patterns and suicidality is valuable for identifying unrecognized suicidality or patients at risk of suicidality. The link between a higher comorbidity burden and increased suicide rates prompts the following question [10-12]: can we use information about a patient’s comorbidity burden to identify patients at risk of suicidality who may be systematically undiagnosed in the electronic health record (EHR)?

Suicidality subtypes are generally diagnosed with respect to severity: ideation, self-harm, and attempt [15,16]. Self-harm, or nonsuicidal self-injury, exists on the same behavioral and psychological continuum as suicidal behaviors and remains one of the strongest predictors of future suicidal behavior [17,18]. The ability to systematically recognize comorbidity-driven risk patterns could facilitate earlier intervention and more accurate surveillance, resource allocation, and cohort identification for research.

Using a large dataset of ED visits from a major academic pediatric hospital, this retrospective study aimed to (1) measure the prevalence of mental health comorbidities in pediatric suicidality and (2) identify undiagnosed high-risk suicidality cases by matching patients with similar mental health comorbidity burden.

## Methods

### Overview

EHR data were extracted and analyzed for the study period (June 1, 2016, to June 1, 2022). The study population included all patients aged 6 to 18 years [15,19] who visited the ED during the study period, identified through the presence of an ED note. Patients were screened for suicide using the Ask Suicide-Screening Questions toolkit [20]. Patient EHR data were deidentified and loaded into Cumulus (Computational Health Informatics Program) [21], a platform for population health studies [22,23].

An *ICD-10*–based suicidality case definition [5] was used to select patient cohorts who matched suicidality criteria. Comorbidities in the suicidality population were reviewed as potential study variables for the *ICD-10*–based suicidality case definition. Statistically significant associated comorbidities were then used as input variables for a propensity score matching (PSM) model to select patients who did not match an *ICD-10*–based suicidality case definition [24]. The propensity-matched cohorts were compiled for chart review. The accuracy of the comorbidity-enriched PSM model was then compared to the interpretation of 2 subject matter experts (RX and AJM).

**Figure 1.**
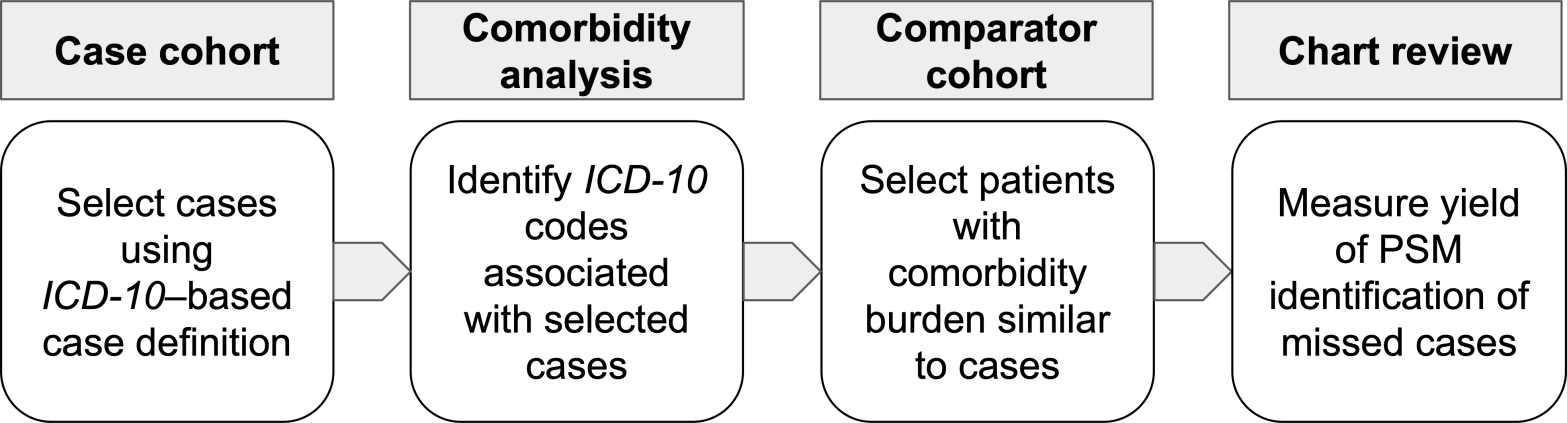
Sequence of case cohort selection, comorbidity analysis, comparator cohort matching, and manual chart review. *ICD-10*: *International Classification of Diseases, 10th Revision*; PSM: propensity score matching.

Figure 1 shows the sequence of case cohort selection, comorbidity analysis, comparator cohort matching, and chart review validation. First, cohorts were selected using an *ICD-10*–based suicidality case definition. Second, suicidality comorbidities were selected using conditional probabilities, tested for significance, and corrected for multiple hypothesis testing. Third, PSM was used to select patients similar to known suicidality cases. Fourth, manual chart review was used to assess the PSM model identification of suicidality cases missed by conventional *ICD-10*–based case selection.

### Ethical Considerations

This retrospective observational study was approved by the Boston Children’s Hospital institutional review board (IRB-P00043211) with a waiver of ethics approval and informed consent. Patient data were deidentified before analysis.

### Case Definition

Diagnosis of suicidality included 3 subtypes with increasing severity: ideation, self-harm, and attempt [6,24]. *ICD-10* codesets were compiled for each suicidality subtype [5]. Suicidal ideation is documented only for the present encounter (R45.851) as there are no *ICD-10* codes for history of suicidal ideation. Self-harm has 4 codes for personal history (R45.88, Z91.5, Z91.51, and Z91.52) and 1396 codes in total. Suicide attempt includes 4 codes: suicide attempt (T14.91), initial encounter (T14.91XA), subsequent encounter (T14.91XD), and sequela of suicide attempt (T14.91XS).

### Study Variables

Study variables included the *ICD-10*–based suicidality case definition, *ICD-10*–based diagnosis codes for comorbid *DSM-5* disorders, patient demographics, encounter service date, health care use, and physician note types. Demographic variables included patient-reported race, sex assigned at birth, and age at ED visit, with age groups categorized as children (6‐11 years) and adolescents (12‐18 years). The encounter service date was used to determine the encounter sequence. Health care use provided a measure of the total number of patient visits and the number of clinical note types per patient. Each encounter was linked to clinical notes including the ED note and, optionally, psychiatric notes and discharge summary. The statistically significant codes resulting from the comorbidity analysis were added to the list of study variables (Multimedia Appendix 1).

### Comorbidity Analysis

Conditional probability was used to quantify the relationships between additional *ICD-10* diagnoses and the *ICD-10*–based suicidality case definition. The conditional probability of suicidality *A* given the concurrent presence of a comorbid condition *B* in the same patient (equation 1) is denoted as follows:


(1) $$P(A|B)=\frac{P(A\cap B)}{P(B)}$$

*A* denotes the suicidality *ICD-10* codeset. *B* denotes a particular *ICD-10* diagnosis not included in the suicidality codeset. *P(A ∩ B)* signifies the probability of suicidality *A* and diagnosis *B* occurring in the same patient, and *P(B)* denotes the independent probability of another *ICD-10* diagnosis code *B*. Expressed within the range of 0 to 1, a value of 0 signifies that the diagnosis pairs never co-occur, whereas a value of 1 indicates that the diagnosis pairs always co-occur. Conditional probabilities were calculated using equation 1 for all possible co-occurring *ICD-10* diagnosis codes stratified by age group, sex assigned at birth, and suicidality subtype (ideation, attempt, and self-harm). To increase the reproducibility and practical utility of the comorbidity model, only the 20 top-scoring co-occurring *ICD-10* codes were selected for each stratification. We then tested the statistical significance of these associations for each stratifier using a chi-square test for independence, with a Bonferroni correction applied to control for false discovery of multiple comparisons (SciPy; version 1.15.2). *ICD-10* codes occurring in fewer than 20 patient cases were omitted from the analysis to reduce the effect of rare events [25]. Conditional probabilities and *ICD-10* diagnosis pairs are further referred to as comorbidities even when not all *ICD-10* codes necessarily indicate a clinically recognized comorbid condition.

### PSM Approach

PSM was used to match the case cohort to a comparator cohort covariates across all study variables, that is, patient demographics (age group, biological sex, and race), health care use codes, clinical note types, *DSM-5* categories, and significant *ICD-10* codes selected from the comorbidity analysis (Multimedia Appendix 1). The case cohort included every patient encounter with a suicidality diagnosis matching the case definition. The comparator cohort included every other patient encounter during the study period excluding patients matching the case definition. Case and comparator cohorts were compiled and ordered by encounter date, aggregating study variable observations up to the date of the encounter. To attain larger sample sizes and reduce variation in *ICD-10* billing practices, *ICD-10* codes were grouped using the *DSM-5*, *Text Revision* [9].

The study variables were compiled into a feature matrix [26] and analyzed using the *PsmPy* Python package [27]. A logistic regression model estimated propensity scores for suicidality using the feature matrix as input. Similarity between suicidality cases and patients without a suicidality diagnosis was measured using the PSM logit score (ie, the logarithmic transformation of the predicted probability and k-nearest neighbor).

The source code [28] for reproducing this work is freely available under an open-source Apache 2.0 License.

## Results

### Cohort Characteristics

During the study period, there were 59,866 patients aged 6 to 18 years with 90,980 ED encounters in total, of which 2638 (2.9%) encounters matched an *ICD-10*–based case definition of suicidality. Table 1 reports demographic characteristics for all ED encounters stratified for each suicidality subtype. Suicidality was significantly more common among female than male individuals across all suicidality subtypes (*P*<.001 via chi-square test).

**Table 1.** Cohort characteristics.

Demographics	All ED^a^ encounters (n=90,980), n (%)	ED encounters—patients with suicidality (n=2638), n (%)	Suicidality subtypes, n (%)		
			Ideation (n=2,275)	Self-harm (n=1030)	Attempt (n=177)
Aged 6-11 years	47,253 (51.9)	310 (11.8)	266 (11.7)	69 (6.7)	5 (2.8)
Aged 12-18 years	43,727 (48.1)	2328 (88.2)	2009 (88.3)	961 (93.3)	172 (97.2)
Male	47,045 (51.7)	813 (30.8)	707 (31.1)	222 (21.6)	37 (20.9)
Female	43,929 (48.3)	1825 (69.2)	1568 (68.9)	808 (78.4)	140 (79.1)

a ED: emergency department.

Table 2 shows the *ICD-10*–based prevalence of mental health conditions observed in all ED encounters according to *DSM-5* categories. Anxiety disorders were the most prevalent (9343/59,866, 15.6%), followed by attention-deficit/hyperactivity disorder (6250/59,866, 10.4%) and depressive disorders (5426/59,866, 9.1%). Notably, gender dysphoria (333/59,866, 0.6%), dissociative disorders (232/59,866, 0.4%), and personality disorders (190/59,866, 0.3%) were rare *DSM-5* categories.

**Table 2.** Prevalence of mental health conditions observed in all emergency department encounters (n=59,866) based on the *Diagnostic and Statistical Manual of Mental Disorders, Fifth Edition*.

Disorder	Prevalence, n (%)
Anxiety disorders	9343 (15.6)
Attention-deficit/hyperactivity disorder	6250 (10.4)
Depressive disorders	5426 (9.1)
Trauma- and stressor-related disorders	5410 (9.0)
Sleep-wake disorders	4927 (8.2)
Disruptive, impulse control, and conduct disorders	3646 (6.1)
Communication disorders	2863 (4.8)
Autism spectrum disorder	2769 (4.6)
Intellectual disabilities	2728 (4.6)
Somatic symptom and related disorders	2614 (4.4)
Other neurodevelopmental disorders	2514 (4.2)
Elimination disorders	1955 (3.3)
Other mental disorders	1803 (3.0)
Medication-induced movement disorders and other adverse effects of medication	1304 (2.2)
Feeding and eating disorders	1256 (2.1)
Motor disorders	1086 (1.8)
Substance-related and addictive disorders	1010 (1.7)
Obsessive-compulsive and related disorders	933 (1.6)
Tic disorders	879 (1.5)
Specific learning disorder	738 (1.2)
Neurocognitive disorders	686 (1.1)
Bipolar disorder	448 (0.7)
Schizophrenia spectrum and other psychotic disorders	411 (0.7)
Gender dysphoria	333 (0.6)
Dissociative disorders	232 (0.4)
Personality disorders	190 (0.3)

### Comorbidity Analysis

**Figure 2.**
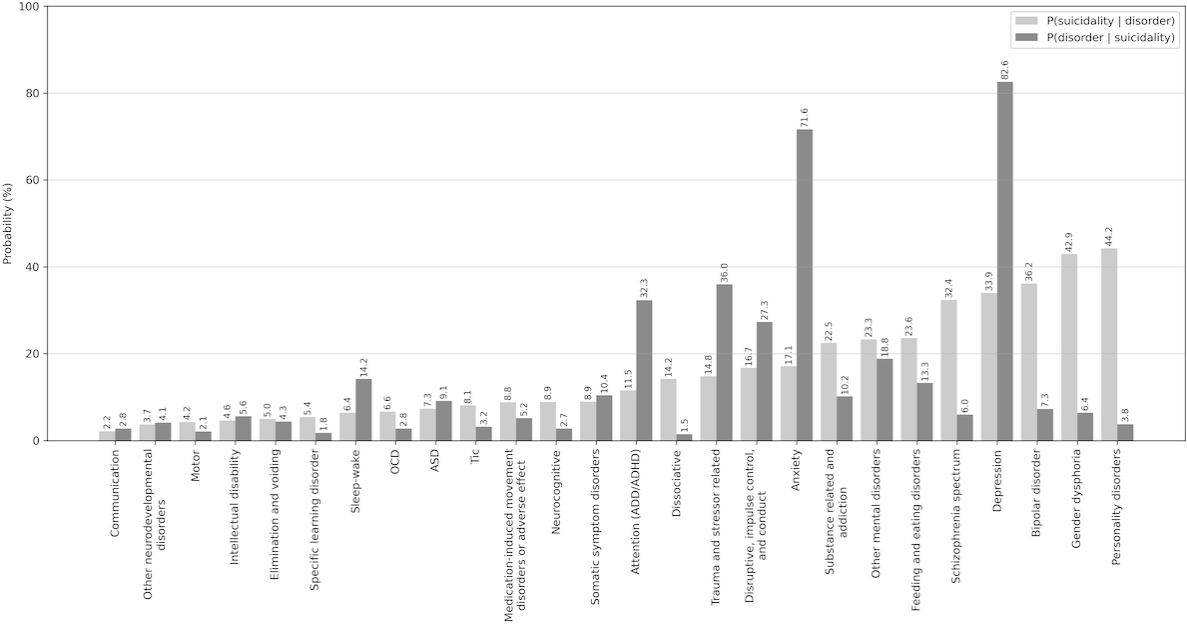
Conditional probabilities of suicidality and *Diagnostic and Statistical Manual of Mental Disorders, Fifth Edition*–categorized mental health conditions. The light gray bars show the probability of suicidality given a specific disorder, whereas the dark gray bars show the probability of a disorder given that individuals also had a documented *International Classification of Diseases, 10th Revision*, code for suicidality. ADD: attention-deficit disorder; ADHD: attention-deficit/hyperactivity disorder; ASD: autism spectrum disorder; OCD: obsessive-compulsive disorder.

Comorbidity analysis revealed that the conditional probability of suicidality was highest for the following *DSM-5* categories: personality disorders (84/190, 44.2%), gender dysphoria (143/333, 42.9%), bipolar disorder (162/448, 36.1%), depression (1842/5426, 33.9%), and schizophrenia spectrum disorders (133/411, 32.4%). Conversely, the probability of *DSM-5* category–given suicidality was highest for depression (1842/2230, 82.6%); anxiety (1597/2230, 71.6%); trauma- and stressor-related disorders (802/2230, 36.0%); attention-deficit disorder and attention-deficit/hyperactivity disorder (721/2230, 32.3%); and disruptive, impulse control, and conduct disorders (609/2230, 27.3%). Nearly half (1127/2230, 50.5%) of all patients with an *ICD-10* code for gender dysphoria (F64.8) also met the suicidality case definition. Figure 2 illustrates the conditional probabilities of suicidality and *DSM-5*–categorized mental health conditions. Detailed statistics for each *DSM-5* category can be found in Multimedia Appendix 2.

Of the 55 statistically significant *ICD-10* suicidality risk factors, nearly half (n=26, 47.3%) were not part of the *DSM-5* (Multimedia Appendix 1). Nearly a quarter (n=12, 21.8%) were for harm without documented self-harm intent, including “unintentional” drug poisoning and “accidental” lacerations of arms and legs. These *ICD-10* codes for unintentional poisoning or accidental injury were strongly associated with self-harm diagnosis. Female patients were significantly more likely (odds ratio 2.23, 95% CI 1.61‐3.10; *P*<.001) than male patients to have *ICD-10* codes related to accidental (unintentional) or undetermined self-harm. Multimedia Appendix 3 provides a more detailed report on findings between subpopulations.

Mental health conditions such as F48.9 (nonpsychotic mental disorder, unspecified), F43.12 (posttraumatic stress disorder, chronic), and R44.0 (auditory hallucinations) were not found in *DSM-5* classifications [9], yet they were significant risk factors for suicidality within the study population. A full list of the top-scoring risk factors for suicidality is provided in Multimedia Appendix 4.

### PSM Results

**Figure 3.**
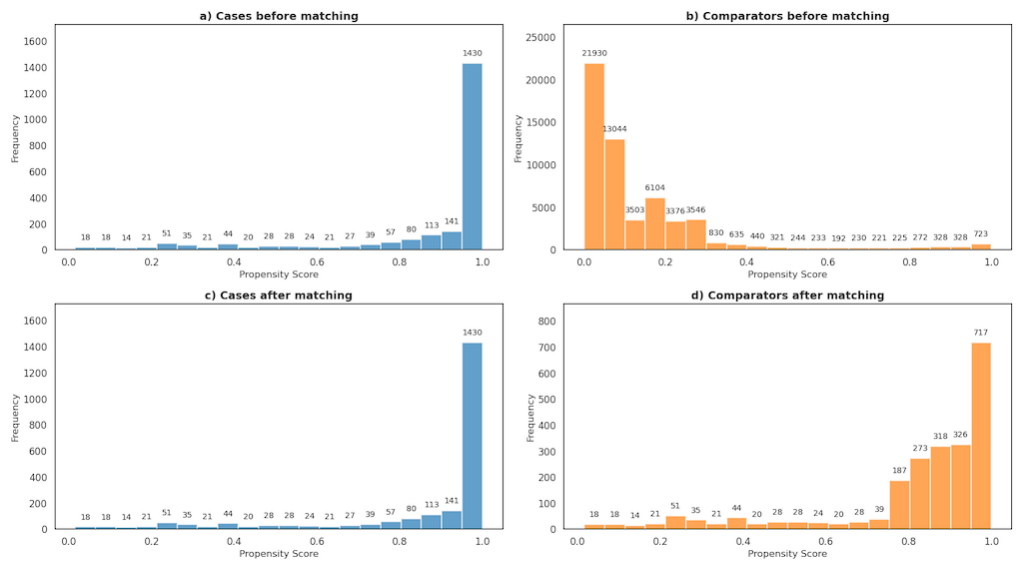
Distribution of propensity scores for suicidality cases and comparators before and after matching. The x-axis denotes propensity scores. The y-axis denotes the frequency, representing the size of the patient population.

PSM was used to match cases with comparators sharing a similar comorbidity burden that may underreport cases using an *ICD-10*–based case selection. Figure 3 illustrates the distribution of propensity scores for suicidality cases and comparators before and after matching. Propensity scores closer to 0 indicate lower likelihood of suicidality, whereas scores closer to 1 represent a higher likelihood. Before matching, 70.4% (1571/2230) of suicidality cases (Figure 3) had a propensity score of ≥0.9. In contrast, only 1.9% (1051/56,725) of comparators had scores of ≥0.9 (Figure 3). After matching, the distribution of matched comparators (Fig. 4d) more closely resembled that of the cases. The number of comparators with propensity scores of <0.5 dropped from 94.7% (53,729/56,725) before matching to 12.2% (270/2230) after matching, demonstrating that PSM was effective in aligning the comparator group’s comorbidity profile with that of the suicidality cases. Multimedia Appendix 5 provides more details on the PSM analysis.

Propensity-matched cohorts were selected for chart review (n=205 encounters) by subject matter experts [5]. Compared to the prevalence of suicidality in the overall study population (2638/90,980, 2.9%), the prevalence in a propensity-matched cohort was 22.3% (23/102) per human expert review. The difference in proportions of suicidality in the 2 populations was significant (*P*<.001; 2-proportion *z* test).

## Discussion

### Principal Findings

In this study, a patient-matching method using comorbidities as covariates identified a cohort of patients with high prevalence of suicidality despite lacking *ICD-10* codes for suicidality. This finding has several important implications. First, the PSM methodology could enable more accurate cohort identification for suicidality research; public health surveillance; and population-level analyses of suicidality risk, prevalence, and outcomes. Currently, the ability to identify suicidality cases relies heavily on *ICD-10* codes for suicidality, which are far from comprehensive and miss many cases [5-8]. Our established methodology incorporated both *DSM-5*–related codes and *ICD-10* codes not related to the *DSM-5*, expanding the range of comorbidity patterns associated with suicidality. Non–*DSM-5* codes, such as accidental poisoning or injury-related encounters, were statistically significant, indicating that relevant risk factors extend beyond formal psychiatric diagnoses. By demonstrating the utility of this broader, comorbidity-informed PSM approach to identify potential underdiagnosis and/or inadequate documentation of suicidality, this study advances methods for improving case ascertainment, including unrecognized cases of suicidality (ie, those that are not labeled with an *ICD-10* code for suicidality yet have demonstrated evidence of suicidal behavior).

These findings have important clinical implications as well. Mental illness and suicidality are a growing problem among youth in the United States. The ability to identify these cases accurately is essential both for clinicians to provide appropriate mental health care and reduce suicidality among those at risk, who may currently go unrecognized, and for researchers to study these problems and generate insights that can inform public health resource allocation and intervention development. This study identified comorbidity patterns that are associated with increased probability of suicidality. In patients with these phenotypes, additional screening may be warranted to assess for suicide risk. For instance, patients with multiple ED encounters for supposedly unintentional self-harm may be at increased risk of suicidality and require more in-depth psychiatric evaluation than the standard suicide screening measures used for all ED patients.

Suicide attempts were identified among preadolescent patients (<12 years), with a prevalence of 2.8% (5/177). The presence of suicide attempts in this younger age group underscores that suicidal behavior can emerge earlier in development than traditionally recognized. This observation is consistent with recent evidence reporting measurable rates of suicidal ideation and attempts in preadolescents [7,10] and emphasizes the need for early detection strategies and age-specific prevention efforts within pediatric clinical care. Notably, subclinical personality pathology in the developmental age (ie, personality disturbances) may amplify the effects of co-occurring psychiatric conditions, contributing additively to suicidality risk [29] and highlighting vulnerabilities that extend beyond what can be captured by categorical diagnostic frameworks such as the *ICD-10* and *DSM-5*. Similar to suicidality, personality pathology may be underrecognized in pediatric populations due to these diagnostic limitations.

Moreover, while the objective of this study was to develop a method to identify unrecognized cases of existing suicidality rather than predict future risk of suicidality [30], it is feasible that the high-risk comorbidity patterns identified in this study are also associated with increased risk of future suicidality. Additional research is needed to understand how different comorbidity patterns predict suicidality risk and how such insights could be operationalized to improve care and outcomes. Future research could also examine how developmental processes interact with conditions such as personality pathology and gender dysphoria in shaping suicidality risk. These interactions may be more accurately captured through dimensional assessment frameworks that evaluate personality functioning and emotional regulation along a continuum rather than through categorical diagnoses [30].

Public health surveillance of pediatric suicidality using *ICD-10* codes is likely underreported. In the chart-reviewed sample, only approximately half (53.4%) of suicidality cases were identified using *ICD-10* codes. Recent pediatric suicidality studies have reinforced the need for computable phenotypes beyond a traditional *ICD-10*–based case definition of strict diagnostic criteria, namely, machine learning [7,31] and natural language processing [32,33] methods. While it was beyond the scope of this study to estimate the true and apparent prevalence [34] of suicidality, it may be possible to use large language models to provide a more accurate prevalence estimation [35].

### Limitations

There were limitations in this retrospective observational study using EHR data. First, this study was conducted at a single large pediatric medical center in a northeastern urban area of the United States. Clinically relevant comorbidity profiles may vary by geography and urban vs rural settings. Second, this study focused on suicidality in ED encounters. *ICD-10* coding practices may vary across ED, inpatient, and ambulatory settings. Third, while PSM identified many instances of suicidality that were missed using *ICD-10* codes alone, it still might not identify all suicidality cases. Fourth, the effectiveness of PSM is highly dependent on the selected covariates [36]. Unmeasured or uncoded diagnoses could not be accounted for, potentially limiting the accuracy of matched comparisons. To maintain clinical interpretability and feasibility, we restricted the model to top-ranking comorbidities that a clinician could reasonably monitor in practice. However, other significant comorbidities not included in the model may also influence suicidality risk and affect matching accuracy. Further studies to test and refine the methodology may help increase the case detection yield.

### Conclusions

This study provides a method for selecting case-matching cohorts with pediatric suicidality. Variables for the matched selection included significantly associated suicidality comorbidities and demographic variables in the study population. These findings suggest that pediatric suicidality may be highly underreported using an *ICD-10*–based case definition of suicidality.

## Supplementary material

10.2196/81499 Multimedia Appendix 1 Significant suicidality risk factors.

10.2196/81499 Multimedia Appendix 2 *Diagnostic and Statistical Manual of Mental Disorders, Fifth Edition*, disorder prevalence and suicidality risk probabilities.

10.2196/81499 Multimedia Appendix 3 Subpopulation findings.

10.2196/81499 Multimedia Appendix 4 Top 20 *International Classification of Diseases, 10th Revision*, codes by sex, age, and suicidality subtype.

10.2196/81499 Multimedia Appendix 5 Propensity score matching.

## Abbreviations

*DSM-5*: *Diagnostic and Statistical Manual of Mental Disorders, Fifth Edition*
ED: emergency department
EHR: electronic health record
*ICD-10*: *International Classification of Diseases, 10th Revision*
PSM: propensity score matching
